# Midterm results of left coronary artery reimplantation through the transverse sinus of the pericardium in adult Bland-White-Garland syndrome

**DOI:** 10.1186/s40792-015-0027-3

**Published:** 2015-03-10

**Authors:** Go Kataoka, Kiyoharu Nakano, Ryota Asano, Atsuhiko Sato, Kojiro Kodera, Wataru Tatsuishi, Shinji Sudo

**Affiliations:** Department of Cardiovascular Surgery, Tokyo Women’s Medical University, Medical Center East, 2-1-10, Nishiogu, Arakawa-ku, 116-8567 Tokyo Japan; Department of Cardiovascular Medicine, Yatsu Hoken Hospital, Chiba, Japan

**Keywords:** Coronary artery disease, Bland-White-Garland syndrome, Adult

## Abstract

The anomalous origin of the left coronary artery from the pulmonary artery - known as Bland-White-Garland syndrome - is a rare congenital malformation that affects 1 in 300,000 live births. Most patients die in infancy without any surgical treatment. Some patients who survive past childhood often have varying symptoms such as myocardial ischemia, impaired left ventricular function, mitral regurgitation, and progressive heart failure, depending on the development collateral circulation. In the present report, we describe a procedure wherein the left coronary artery ostium was translocated through the transverse sinus of the pericardium in a 43-year-old mother with Bland-White-Garland syndrome and concomitant mitral regurgitation and report on the associated midterm results.

## Background

The anomalous origin of the left coronary artery from the pulmonary artery (ALCAPA) occurs in 0.26% of patients with congenital heart disease. Up to 90% of ALCAPA patients die during their first year of life because of left ventricular failure in cases where it is surgically corrected. Adult presentation of ALCAPA is rare but may occur in cases where a well-developed collateral circulation from the right coronary artery is formed. Adults with ALCAPA are at considerable risk of chronic ischemic congestive heart failure and sudden death [1]. We describe a modified procedure for the translocation of the left coronary artery (LCA) combined with mitral annuloplasty in an adult with ALCAPA and concomitant mitral regurgitation (MR) and the associated midterm results.

## Case presentation

A 43-year-old mother (of two children) had a heart murmur since childhood; however, due to the absence of symptoms, no action was taken. Her pregnancies and deliveries were normal. However, she started to experience palpitations and shortness of breath after approximately 1 year.

**Figure 1:**
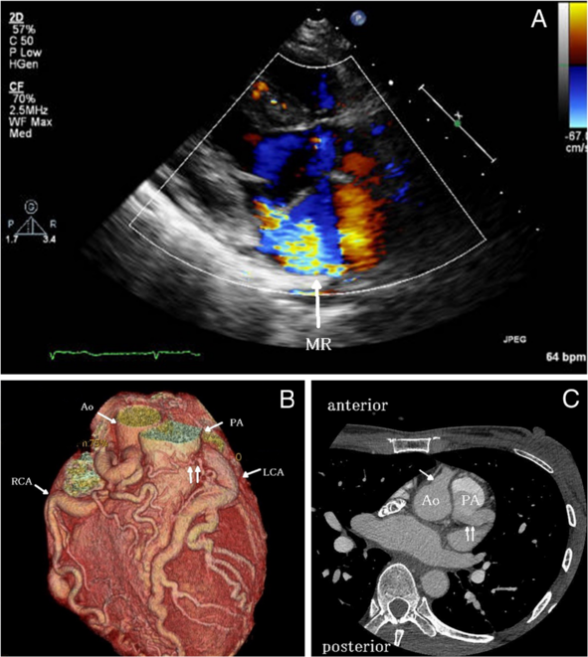
**Results of diagnostic examination. (A)** Long-axis view on preoperative transthoracic echocardiography showing moderate or severe mitral regurgitation. **(B)** Preoperative multidetector-row computed tomography. The RCA and the LCA were dilated, and collaterals between the two coronary arteries were observed. **(C)** Preoperative axial image obtained via enhanced computed tomography. The arrow indicates the RCA orifice, and the double arrows indicate the LCA orifice. The LCA originated from the PA. The orifice of the LCA was located 1.8 cm from the Ao. Ao, aorta; PA, pulmonary artery; RCA, right coronary artery; LCA, left coronary artery.

Physical examination indicated a regular pulse of 70 beats/min, blood pressure of 120/60 mmHg, and a pansystolic murmur at the left lower part of the sternum. Chest radiography showed a cardiothoracic ratio of 50%. The electrocardiogram showed a sinus rhythm with left axis deviation, ST depression in V_4_ to V_6_, and a negative T in aVL and V_1_ to V_3_. Ambulatory monitoring revealed non-sustained ventricular tachycardia. Transthoracic echocardiography demonstrated a good ejection fraction (64%); however, the anterior wall motion of the left ventricle indicated mild hypokinesis. The left ventricular end-diastolic/end-systolic dimension (LVDd/Ds) value was 64/41 mm, and moderate-to-severe MR was noted (Figure 1A). Echo examination showed a dilated right coronary artery (RCA) originating from the ascending aorta; however, the origin of the LCA could not be determined.

Coronary angiography confirmed that the dilated and tortuous RCA arose from the aorta, although the origin of the LCA could not be identified. Collateral flow from the RCA to the LCA and pulmonary trunk was confirmed. However, pulmonary hypertension was not present (systolic/diastolic pressure, 21/9 mmHg). Multidetector-row computed tomography (MDCT) indicated the development of collaterals between the RCA and the LCA, RCA dominance, and a hypoplastic circumflex artery (CX) and also indicated that the LCA arose from the pulmonary trunk and that the dilated RCA originated from the appropriate sinus of Valsalva (Figure 1B). The orifice of the LCA was located 1.8 cm from the aorta (Figure 1C). The patient was diagnosed with ALCAPA with concomitant moderate-to-severe MR.

**Figure 2:**
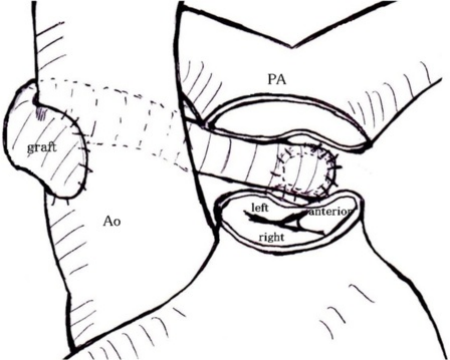
**Operative schema.** The PA was transected. The LCA ostium was cut out from the posterior PA wall to obtain a Carrel patch, with a cuff diameter of 8 mm. A graft was anastomosed to the coronary cuff with 5–0 polypropylene sutures. The graft was directed toward the right lower side of the aorta through the transverse sinus of the pericardium, and its proximal side was then anastomosed to an opening prepared on the right lateral side of the ascending Ao with 5–0 polypropylene sutures. The PA was directly reconstructed with 4–0 polypropylene sutures. Ao, aorta; PA, pulmonary artery; RCA, right coronary artery; LCA, left coronary artery. Graft: an 8-mm HEMASHIELD graft (MAQUET Cardiovascular LLC, San Jose, CA, USA); anterior, anterior semilunar cusp; left, left semilunar cusp; right, right semilunar cusp.

Surgery was performed via a median sternotomy. Extracorporeal circulation was established by aortic cannulation and bicaval drainage through the superior vena cava and the right atrium under moderate hypothermia; the left heart structures were vented via the right upper pulmonary vein. Myocardial protection was achieved by anterograde cold cardioplegia after cross-clamping. The main pulmonary trunk was incised transversely. The LCA ostium was identified at the left-facing sinus of the pulmonary valve, adjacent to the commissure at the non-facing sinus. Collaterals were noted around the left main trunk, and the LCA ostium was located far from the aorta; therefore, aggressive mobilization of the left main trunk was not considered to be sufficient to enable direct implantation of the LCA directly at the aorta. The ostium was cut out to obtain a Carrel patch, with a cuff diameter of 8 mm. An 8-mm HEMASHIELD graft (HEMASHIELD, MAQUET Cardiovascular LLC, San Jose, CA, USA) was anastomosed to the coronary cuff with 5–0 polypropylene sutures. The graft was directed toward the right lower side of the aorta through the transverse sinus of the pericardium, while care was taken to avoid kinking or damage; its proximal side was then anastomosed to an opening prepared in the right lateral side of the ascending aorta with 5–0 polypropylene sutures (Figure 2).

Right-sided left atriotomy was then performed. The mitral leaflets were thickened, but there was no prolapsed lesion. Mitral annuloplasty was performed using a Carpentier-Edwards Physio II 30-mm ring (Edwards Lifescience Ltd., Irvine, CA, USA), with no residual leakage. The atriotomy was closed, and the pulmonary trunk was directly reconstructed with 4–0 polypropylene sutures. Transesophageal echocardiography demonstrated no MR. The period of weaning from extracorporeal circulation was uneventful, and the postoperative course was straightforward.

**Figure 3:**
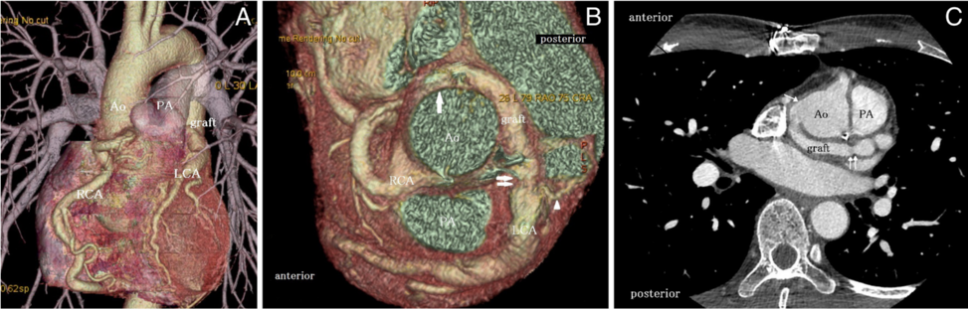
**Computed tomography exam results in different perspectives. (A)** Multidetector-row computed tomography 2 years after surgery. The diameter of the preoperatively dilated coronary artery reduced and the collaterals disappeared. **(B)** Cranial view on multidetector-row computed tomography at 2 years after surgery. The arrow indicates that the graft was anastomosed to the right lateral side of the ascending Ao. The double arrows indicate that the graft was anastomosed to the LCA. The arrowhead indicates the collateral located adjacent to the anastomotic site of the graft and the LCA. **(C)** Axial image on enhanced computed tomography at 2 years after surgery. The arrow indicates that the graft was anastomosed to the right lateral side of the ascending Ao. The double arrows indicate that the graft was anastomosed to the left coronary cuff. Ao, aorta; PA, pulmonary artery; RCA, right coronary artery; LCA, left coronary artery. Graft: an 8-mm HEMASHIELD graft (MAQUET Cardiovascular LLC, San Jose, CA, USA).

At 2 years after surgery, no MR or pulmonary trunk stenosis was noted, the LVDd/Ds value improved to 47/25 mm, and the ejection fraction increased to 77%, without any hypokinesis in the left ventricular anterior wall on transthoracic echocardiography. The ST depression in V4 to V6 and a negative T in aVL and V1 to V3 that were noted on electrocardiography had normalized. The diameter of the preoperatively dilated coronary artery reduced, and the developed collaterals had disappeared (Figure 3A). MDCT indicated that the graft was patent with no kinking (Figure 3B, C). The patient’s dyspnea had improved by one NYHA class, without any anticoagulation.

ALCAPA is rarely seen in adults. Without surgical intervention, malignant ventricular arrhythmias and sudden death - secondary to myocardial ischemia and global cardiomyopathy - will occur with an estimated incidence of 80% to 90% at a mean age of 35 years [2]. On the other hand, Nightingale et al. reported two cases of middle-aged mothers with ALCAPA presenting good tolerance and long-term survival without surgery [3]. In the present case, we believe that the reason why the adult patient showed good tolerance even during pregnancy and after giving birth may include the development of collateral circulation from the RCA to the LCA; a sufficient blood supply to the LCA lesion, with an RCA-dominant anatomy, and a hypoplastic CX from the early postnatal period; and minimal coronary flow from the RCA through the LCA into the pulmonary trunk. Furthermore, a good ejection fraction was maintained, and pulmonary hypertension was not observed even in adulthood. However, we believe that she exhibited mild hypokinesis of the anterior LV wall, left ventricular dilatation, and moderate-to-severe MR caused by papillary muscle dysfunction because the chronic myocardial ischemia progressed with a gradual increase in the coronary flow from the RCA through the LCA into the pulmonary trunk. Hence, we decided to perform a surgical intervention to prevent chronic ischemic congestive heart failure, malignant arrhythmia, and sudden death.

The establishment of a two-coronary system is currently the standard method for the repair of ALCAPA in adults [4]. This can be achieved via various techniques, such as direct implantation of the LCA into the aorta, translocation with an extension using various materials, LCA reconstruction using an autologous pulmonary artery, the intrapulmonary technique, and aortocoronary bypass with LCA ligation [5-7]. Due to the lack of sufficient data on adult Bland-White-Garland syndrome, it is unclear which technique is the most suitable. In the present case, we could not aggressively mobilize the left main trunk as collaterals had developed around the left main trunk (Figure 3B); moreover, as the LCA ostium was located far from the aorta (Figure 1C), we did not perform direct implantation of the LCA.

The saphenous vein was not considered to be sufficient for extending the left main trunk to the aorta, as its diameter and the supply of blood flow to the dilated LCA would be inadequate. Moreover, as the pulmonary arterial trunk was not sufficiently dilated, the creation of an intrapulmonary tunnel would cause pulmonary stenosis. Therefore, we chose to perform translocation of the LCA, using a vascular graft, through the transverse sinus of the pericardium. This technique has some advantages: it is simpler than the others, the graft tension can be controlled, and the proximal anastomosis is very easy because the graft is on the right side of the aorta and not behind it. It is similar to Svensson’s modified Bentall technique with a long interposed graft to the LCA [8]. Nakahira et al. had indicated that Svensson’s technique was associated with favorable midterm outcomes by using multislice computed tomography and consecutive echocardiographic evaluations; moreover, this method yielded long-lasting advantages as well as technical benefits [9]. In the present case, the vascular graft was patent without any anticoagulation during 2 years after this operation. We believe that this technique can prevent kinking and overstretching of the repaired LCA and may be related to a long patency, as compared to that achieved by Svensson’s modified Bentall technique.

The establishment of a two-coronary system with conservative management of even severe MR has yielded good results in infants with ALCAPA [10]. However, the possibility of an improvement in left ventricular function after surgical intervention is lower in adult ALCAPA patients than in infants. Therefore, moderate or severe MR in adults with ALCAPA should be resolved [4].

## Conclusions

We have described a case of translocation of the LCA through the transverse sinus of the pericardium, combined with mitral annuloplasty, in a 43-year-old mother (of two children) with ALCAPA and moderate-to-severe MR. We believe that this modified procedure for the translocation of an LCA is one of the reasonable surgical treatments for ALCAPA in adults.

## Consent

Written informed consent was obtained from the patient for publication of this case report and any accompanying images.

## Abbreviations

ALCAPA: anomalous origin of the left coronary artery from the pulmonary artery
CX: circumflex artery
LCA: left coronary artery
LVDd/Ds: left ventricular end-diastolic/end-systolic dimension
MDCT: multidetector-row computed tomography
MR: mitral regurgitation
RCA: right coronary artery
